# Experience in the treatment of giant orbital intraconal teratoma: A case report

**DOI:** 10.1097/MD.0000000000041205

**Published:** 2025-01-03

**Authors:** Ruimiao Li, Mingyu Ren, Limin Liu, Ruina Zhang, Wensheng Guo

**Affiliations:** a Department of Orbital Disease and Ocular Tumor, Hebei Eye Hospital, Xingtai, Hebei, China; b Department of Ophthalmology, the 940 Hospital of Joint Service Support Forces of the Chinese People’s Liberation Army, Lanzhou, China.

**Keywords:** case report, diagnose, orbital teratoma, surgical therapy

## Abstract

**Rationale:**

Orbital teratoma is a relatively rare disease in infancy. In the pediatric population undergoing significant growth and development, the presence of giant orbital masses can significantly affect orbital volumes and impair the visual function. Traditional treatments may not yield anticipated effectiveness, often leading to numerous complications. This report aims to present a rare case involving a giant orbital intraconal teratoma. The objective is to analyze the treatment course comprehensively, distill valuable experiences and lessons learned, enhance treatment strategies, and ultimately reduce the incidence of complications associated with these formidable pediatric tumors.

**Patient concerns:**

A 2-year-old female child was presented exhibiting proptosis and inward and upward eyeball displacement. Enhanced magnetic resonance imaging revealed a well-circumscribed mass, persisting with hypointense signals on T_1_-weighted images (T_1_WI) and hyperintense signals on T_2_-weighted images (T_2_WI).

**Diagnoses:**

The diagnosis of teratoma was confirmed finally through histological and immunohistochemical exams.

**Interventions:**

A transconjunctival approach via the inferior fornix, coupled with canthotomy and cantholysis, was performed. However, a month postsurgery, the patient developed enophthalmos, conjunctival hyperemia, and keratitis upon ocular examination. A second operation involved the implantation of allogeneic sclera into the orbit to increase orbital volume, improve the pitting of the fossa, and restore keratitis to normal.

**Outcomes:**

No recurrence and other complications were noted during the 1-year follow-up.

**Lessons:**

Giant orbital teratomas in children are infrequent and pose significant challenges in the field of therapy. The potential consequences of larger orbital masses in childhood, including increased orbital volume and the risk of postoperative enophthalmos and keratitis. The findings emphasize the importance of timely implantation into the orbital after mass excision to enhance orbital volume and reduce the incidence of complications.

## 1. Introduction

Teratomas, arising from pluripotent germ cells that abnormally migrate to various body parts,^[1]^ represent a rare tumor type composing tissues derived from all 3 germinal layers, existing variable levels of maturity.^[2]^ Classified as congenital, eratomas can manifest as either cystic or solid. Typically, cystic teratomas are mature and benign, and solid variants tend to be immature and malignant.^[3]^ Although teratomas can occur in various anatomical locations, the ovary is the most common site, followed by the testis, sacrococcygeal, retroperitoneal, and mediastinal regions.^[4]^ Notably, teratomas rarely occur in the orbital region, with head and neck teratomas accounting for 0.47% to 6% of reported cases in the literature.^[5]^ Orbital teratomas often present at birth or become evident within the first 6 months of life.^[6]^ Given the dynamic growth and development stage of children, the presence of giant orbital masses can significantly affect orbital volumes, rendering conventional treatments less effective than anticipated and leading to a relatively high incidence of complications. Therefore, this article aims to present a rare case involving a giant orbital intraconal teratoma in the orbital region of a child. The objective is to analyze the treatment course comprehensively, distill valuable experiences and lessons learned, enhance treatment strategies, and ultimately reduce the incidence of complications associated with these formidable pediatric tumors.

## 2. Case report

**Figure 1.**
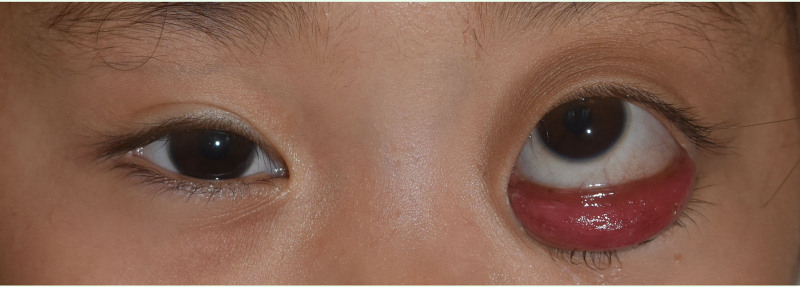
This is a pre-op picture of the patient.

**Figure 2.**
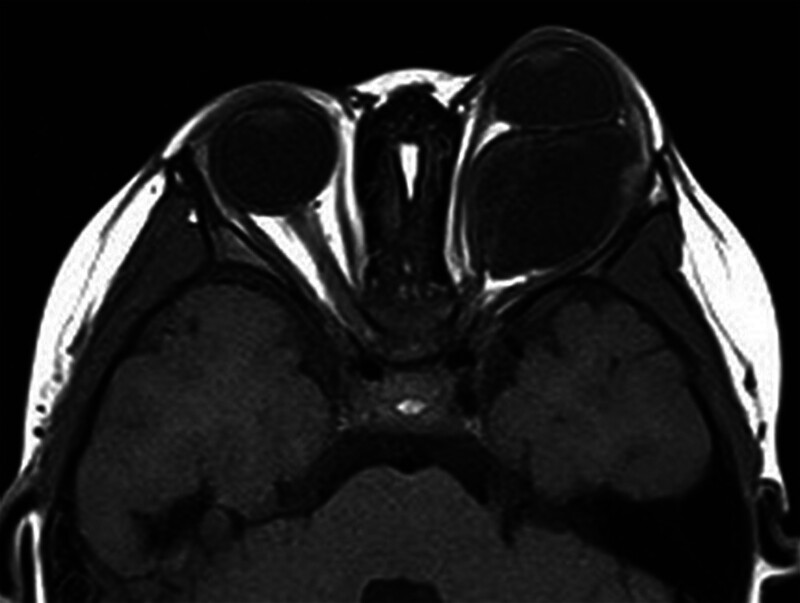
MRI depicting the lesion with hypointense signals on T1-weighted images (T_1_WI) and hyperintense signals on T2-weighted images (T_2_WI). MRI = magnetic resonance imaging, T_1_WI = T_1_-weighted images, T_2_WI = T2-weighted images.

**Figure 3.**
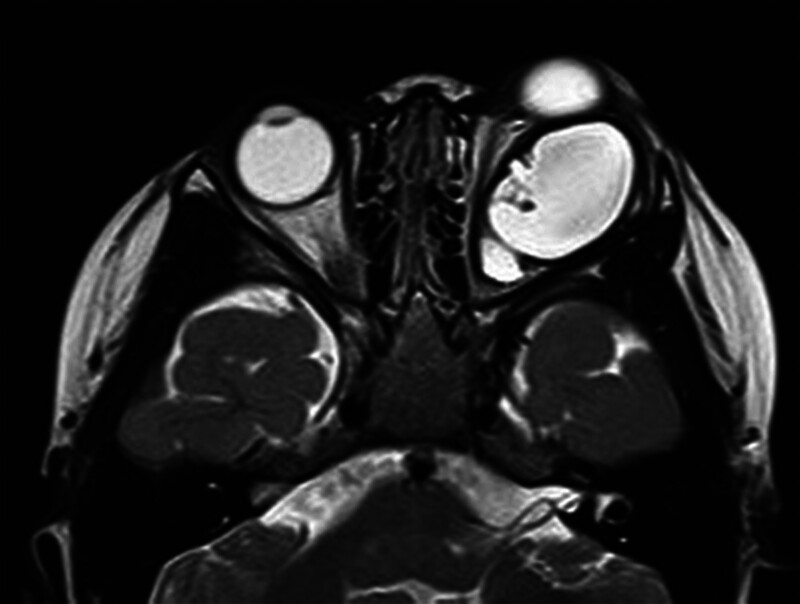
T2-weighted images (T_2_WI) highlighting hyperintense signals within the lesion. T_2_WI = T2-weighted images.

**Figure 4.**
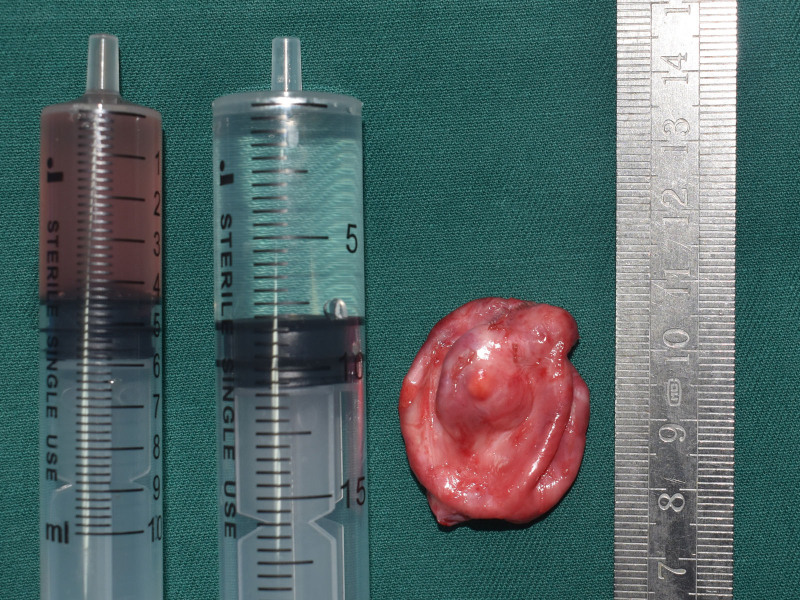
Intraoperative view illustrating the aspiration of approximately 12.5 mL of fluid from the cyst.

**Figure 5.**
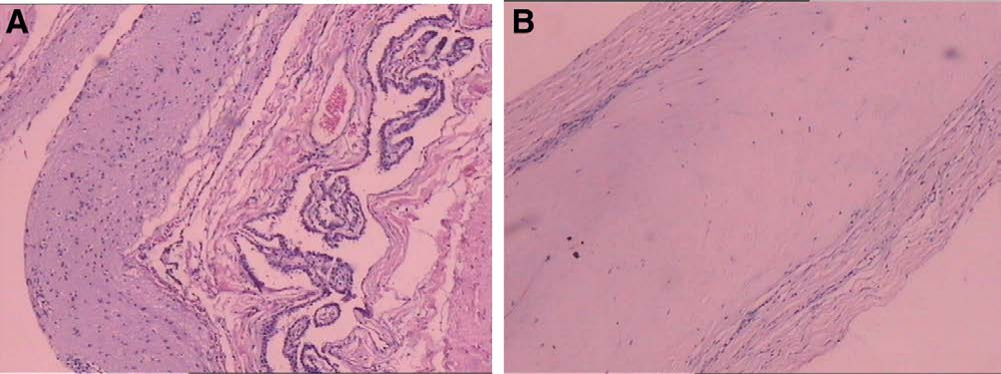
(A) Histopathological examination reveals the fibrous capsule wall covered with glandular epithelium, along with visible brain tissue. (B) Histopathological examination displaying a cartilage-like matrix within the lesion.

**Figure 6.**
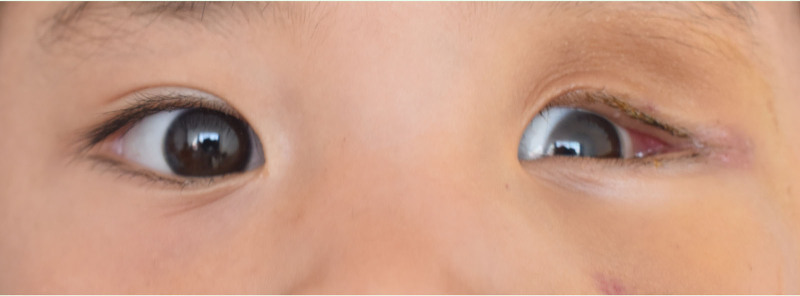
Postoperative image of the patient following the first operation.

**Figure 7.**
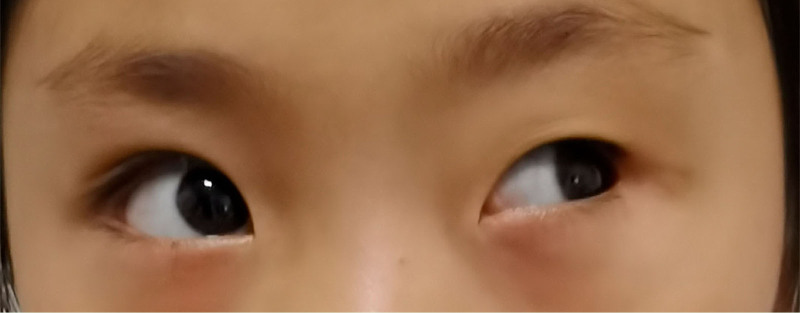
Postoperative image of the patient after the second operation.

A 2-year-old female presented with a 1-year history of painless left progressive proptosis with no reported systemic diseases or family history (Fig. 1). Ophthalmologic examination revealed light sensation as the only vision in the left eye, along with proptosis, inward and upward eyeball displacement, and restricted extraocular muscle movements in downward and outward directions. An irregularly shaped, well-defined soft mass was palpable in the inferior aspect of the left orbit, accompanied by left lower eyelid ectropion. The pupil was enlarged (4 mm in diameter), and pupillary reaction was absent. The remaining anterior segment examination showed no apparent abnormalities. Fundus examination was challenging due to the child’s size. Hertel exophthalmometry readings measured 10.5 mm in the right eye and 18 mm in the left. Magnetic resonance imaging (MRI) revealed a well-circumscribed mass, displaying hypointense signals on T_1_-weighted images and hyperintense signals on T_2_-weighted images (Figs. 2 and 3). Contrast-enhanced imaging demonstrated no significant improvement. A transconjunctival approach via the inferior fornix with canthotomy and cantholysis was performed, revealing a grayish-white cystic mass with a distinct boundary from surrounding tissues. During posterior separation to the eyeballs’ posterior part, tight adhesion to the optic nerve was observed. Due to the mass’s substantial size and the restricted surgical field, volume reduction was necessary. Approximately 12.5 mL of the fluid was aspirated, and the mass was completely excised (Fig. 4). Histopathological examination disclosed a fibrous capsule wall covered with squamous and glandular epithelium, along with visible brain tissue and a cartilage-like matrix consistent with orbital teratoma (Fig. 5A, B). One month postsurgery, the patient exhibited enophthalmos, conjunctival hyperemia, and keratitis on ocular examination. This was attributed to the mass’s prior enlargement of the orbital cavity, resulting in postoperative enophthalmos. The cornea could not adhere to the eyelids, creating a space and causing corneal inflammation (Fig. 6). After obtaining the consent of the patient’s guardian, a second operation involved the implantation of an allogeneic sclera into the orbit to increase the orbital volume, alleviate fossa pitting and restore keratitis to normal (Fig. 7). No recurrence of the teratomas was noted during the 1-year follow-up. The patient still had minor enophthalmos and outer canthus abnormality. The visual acuity remained consistent with pre-operation levels. Hertel exophthalmometry readings measured 10.5 mm in the right eye and 8 mm in the left. The remaining anterior segment examination showed no apparent abnormalities.

## 3. Discussion

Teratomas comprise 6.6% of childhood tumors, commonly occurring in the testes, ovaries, and retroperitoneum.^[7,8]^ Although rare, the orbit can serve as an unusual site for these tumors,^[9–11]^ presenting with clinical features such as severe unilateral exophthalmos, abnormal palpebral fissure, bulbar conjunctival edema, and eyeball displacement. Compression of the eye can lead to corneal exposure and visual loss. When the mass contains bone or cartilage tissue, specific imaging characteristics emerge. Computed tomography scans revealed a well-defined cystic mass with focal bone-like structures or calcific densities in their lumens. Ultrasonic and MRI scans display a cystic mass with a high internal echo within the cyst. However, when the mass lacks bone or cartilage tissue, the imaging findings become less distinctive, posing a diagnostic challenge. The absence of specific clinical manifestations and imaging features often leads to confusion with other orbital cystic lesions, including dermoid cysts, epidermoid cysts, congenital cystic eyes, parasitic cysts, meningoceles, and cystic changes in schwannomas. The gold standard for a definitive diagnosis of teratomas is a pathologic examination. In the case of the child, the pathological analysis revealed the presence of stratified squamous epithelium from the ectoderm and brain tissue from the neuroectoderm; cartilage from the mesoderm; and glandular epithelium from the endoderm. Immunohistochemical results further supported the diagnosis, with positive staining for CKpan (+), CAM5.2 (+), and ki67 (+, <5%). The combined findings from histopathology and immunohistochemistry confirmed the presence of a teratoma.

The primary treatment for teratomas involves the surgical removal of the diseased tissue. However, due to the typically large size of the tumor and the limited space within the orbital surgical field, achieving complete resection of the tumor can be exceptionally challenging. To address this, an initial step during the operation involves aspirating fluid from a giant cyst to reduce the mass size and facilitate complete removal. Previous studies have demonstrated the effectiveness of aspirating the liquid component and replacing it with fibrin glue. This approach aids in the sclerosis of small adjacent vessels, minimizing the risk of bleeding and also causing rupture of the surrounding epithelium, thereby increasing the chances of wholly and radically removing the lesion.^[12–14]^ Despite these efforts, a 1-month postoperative review revealed complications such as enophthalmos, conjunctival hyperemia, and keratitis, likely attributed to the increased size of the mass causing orbital cavity enlargement and postoperative eye concavity. To address these issues, a second operation was conducted involving the implantation of an allogeneic sclera into the orbit. This intervention aimed to increase the orbital volume, improve the pitting of the fossa, and restore keratitis to normal. In the process of selecting an appropriate orbital implant, a comprehensive review of the literature revealed various options, including autologous dermal fat grafts, hydroxyapatite, Medpor plates, and titanium mesh, among others.^[15,16]^ These alternatives have been widely employed in clinical practice, each carrying distinct advantages and disadvantages. Autologous dermal fat grafts have received acclaim for their efficacy; however, their application necessitates the extraction of fat from the periumbilical region. While this technique has proven successful in numerous implants, it comes with the inherent challenge of obtaining donor tissue. Considering the unique circumstances of the case, the choice was made to utilize allogeneic sclera as the preferred implant. This decision was motivated by the implant’s ability to address enophthalmos without introducing additional trauma to the patient. Moreover, the availability of allogeneic sclera from our unit’s eye bank played a pivotal role in influencing this choice. However, it is generally believed that the part of allogeneic sclera is likely to be absorbed by the body. Due to short-term follow-up, the possibility of enophthalmos in the future cannot be ruled out.

In conclusion, giant orbital teratomas in children are rare, and their presence may lead to increased orbital volume and potential postoperative complications such as enophthalmos and keratitis. To mitigate these risks, timely implantation into the orbit after excising the mass and defining its nature is crucial. This clinical case underscores the importance of individualized treatment strategies to optimize outcomes and reduce the incidence of complications in such challenging cases.

## Author contributions

**Conceptualization:** Ruimiao Li, Mingyu Ren.

**Data curation:** Ruimiao Li, Limin Liu.

**Formal analysis:** Mingyu Ren, Limin Liu.

**Investigation:** Limin Liu, Ruina Zhang, Wensheng Guo.

**Methodology:** Ruimiao Li, Mingyu Ren, Limin Liu.

**Project administration:** Ruimiao Li.

**Supervision:** Ruimiao Li, Mingyu Ren.

**Writing – original draft:** Ruimiao Li, Mingyu Ren.
